# Noninvasive indices for predicting nonalcoholic fatty liver disease in patients with chronic kidney disease

**DOI:** 10.1186/s12882-020-01718-8

**Published:** 2020-02-17

**Authors:** A Reum Choe, Dong-Ryeol Ryu, Hwi Young Kim, Hye Ah Lee, Jiyoung Lim, Jin Sil Kim, Jeong Kyong Lee, Tae Hun Kim, Kwon Yoo

**Affiliations:** 1 Division of Gastroenterology and Hepatology, Department of Internal Medicine, College of Medicine, Ewha Womans University, Seoul, Republic of Korea; 2 Division of Nephrology, Department of Internal Medicine, College of Medicine, Ewha Womans University, Seoul, Republic of Korea; 3 Clinical Trial Center, Ewha Womans University Mokdong Hospital, Seoul, Republic of Korea; 4 Department of Radiology, College of Medicine, Ewha Womans University, Seoul, Republic of Korea

**Keywords:** Chronic kidney disease, Nonalcoholic fatty liver disease, Noninvasive markers, Prediction model

## Abstract

**Background:**

Data on clinical characteristics of nonalcoholic fatty liver disease (NAFLD) in patients with chronic kidney disease (CKD) are scarce. We investigated the clinical features and risk factors of NAFLD using noninvasive serum markers in CKD patients and attempted the temporal validation of a predictive model for CKD based on NAFLD.

**Methods:**

This retrospective cross-sectional study was conducted in a single tertiary center. We enrolled 819 CKD patients and evaluated the predictive performance of relevant clinical and laboratory markers for the presence of NAFLD in both derivation (data from 2011 to 2014, *n* = 567) and validation (data from 2015 to 2016, *n* = 252) groups.

**Results:**

In the derivation group, NAFLD was observed in 89 patients (15.7%; mean body mass index (BMI), 24.6 kg/m^2^; median estimated glomerular filtration rate (eGFR), 28.0 ml/min). BMI, hemoglobin, serum alanine aminotransferase, eGFR, and triglyceride-glucose index were used to derive a prediction model for the presence of NAFLD. Using the cutoff value of 0.146, the area under the receiver operating characteristic curve (AUROC) for the prediction of NAFLD was 0.850. In the validation group, NAFLD was observed in 51 patients (20.2%; mean BMI, 25.4 kg/m^2^; median eGFR, 36.0 ml/min). Using the same prediction model and cutoff value, the AUROC was 0.842. NAFLD prevalence in CKD patients was comparable to that in the general population, increasing over time.

**Conclusions:**

Our model using BMI, renal function, triglyceride-glucose index, serum alanine aminotransferase, and hemoglobin accurately predicted the presence of NAFLD in CKD patients.

## Background

Given that its global prevalence is more than 10% of the adult population,chronic kidney disease (CKD) is a global health problem. CKD is a more serious problem in developed countries such as the United States, where the prevalence exceeds 25% of people above 65 years [1]. Because CKD is associated with high morbidity, mortality, and healthcare costs, studies have recently focused on identifying new modifiable risk factors [2]. Nonalcoholic fatty liver disease (NAFLD) is also a growing health problem worldwide, affecting up to one-third of the adult population [3, 4]. Moreover, there is increasing evidence to suggest that NAFLD is a multisystemic disease that affects multiple extrahepatic organ systems such as the cardiovascular system, endocrine organs, and kidneys [5]. Of these multisystemic manifestations, a putative mechanistic link between NAFLD and CKD in terms of pathogenesis has been postulated because NAFLD, metabolic diseases, cardiovascular diseases, and CKD share many metabolic features and risk factors [6]. The key pathophysiological factor in NAFLD is insulin resistance. Insulin resistance leads to triglyceride (TG) deposition in the liver, making it more susceptible to inflammation and fibrosis [7]. Specifically, prediabetic and diabetic patients showed a more severe grade of fat infiltration. This could be due to increased insulin resistance with accompanying dysregulation of lipid metabolism [8]. A series of cross-sectional studies reported that NAFLD is associated with an increased prevalence of CKD, ranging from approximately 20 to 25% [9–11]. Therefore, patients with NAFLD require screening for CKD [12].

NAFLD is an independent risk factor for CKD and is also the most rapidly increasing indication for simultaneous liver-kidney transplantation [13]. Additionally, CKD may exacerbate NAFLD due to altered gut microbiota and barrier function, changed glucocorticoid metabolism, and the accumulation of toxic uremic metabolites [14]. Collectively, it seems logical to evaluate the presence and severity of NAFLD in CKD patients. However, the detailed bidirectional relationship in terms of the pathophysiology and clinical features of NAFLD and CKD is still unclear. Studies on the clinical features of NAFLD in patients with CKD are particularly scarce in the literature. The present study aimed to develop and validate a predictive model for NAFLD in CKD patients based on relevant clinical characteristics.

## Methods

### Patients

**Fig. 1:**
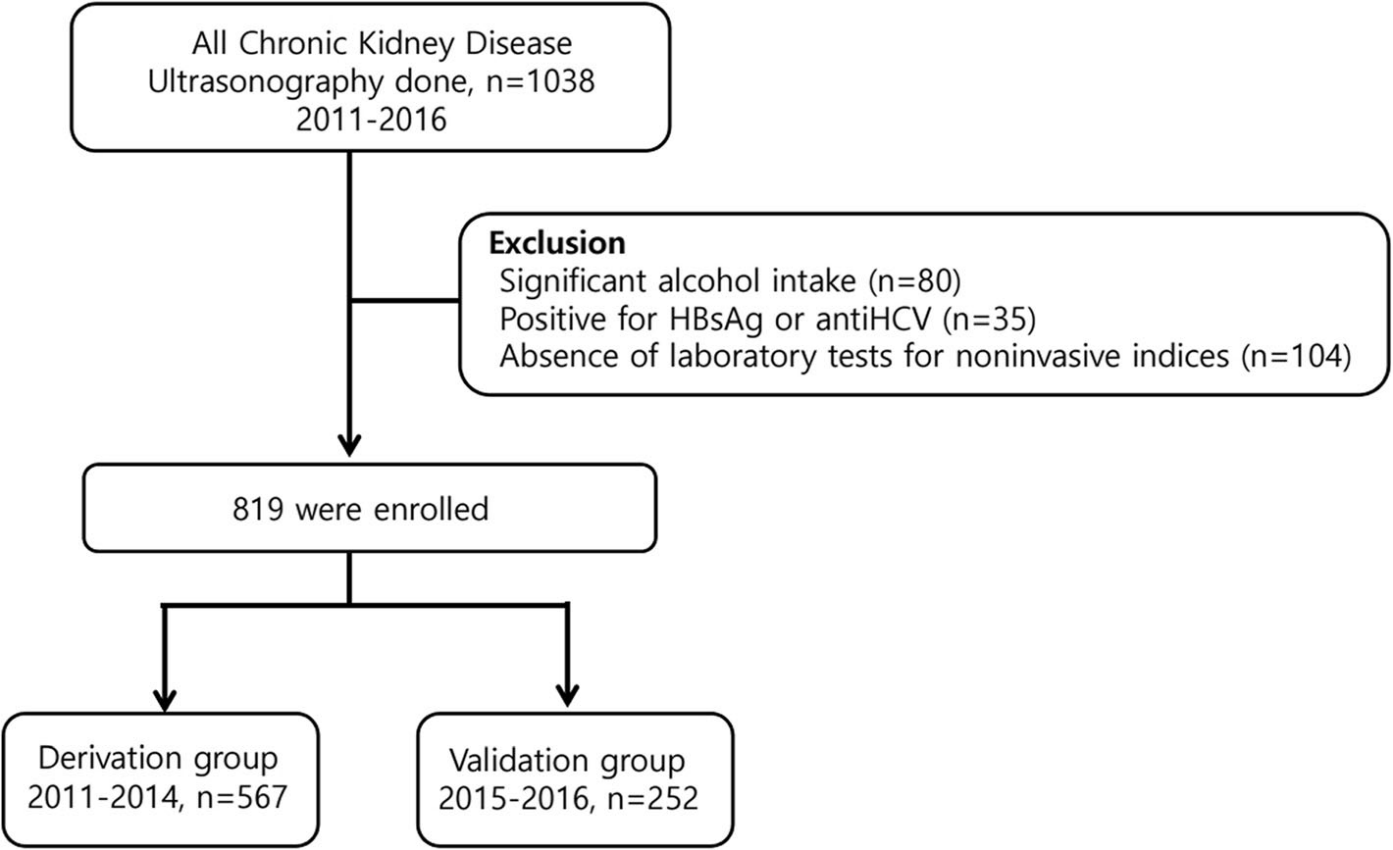
Patients selection diagram

Between January 2011 and December 2016, 1038 patients with CKD were screened for eligibility. The patients had visited the nephrology clinic more than once and underwent abdominal ultrasound as a baseline radiologic evaluation at a university hospital in South Korea. CKD was defined as eGFR < 60 ml/min/1.73 m^2^ according to the Kidney Disease Improving Global Outcomes (KDIGO) guidelines [15]. The exclusion criteria for this study were: (1) age < 18 years, (2) significant alcohol intake (≥30 g/day in men and 20 g/day in women), (3) positivity for hepatitis B surface antigen (HBsAg) or hepatitis C virus antibodies (anti-HCV), (4) presence of other chronic liver diseases such as viral hepatitis, drug-induced liver disease, or autoimmune liver diseases and (5) absence of laboratory tests to calculate noninvasive indices, including triglyceride-glucose index, hepatic steatosis index, NAFLD fibrosis score, BARD score, aspartate aminotransferase/alanine aminotransferase ratio, and fibrosis-4 (FIB-4) score. Of these 1038 patients, 219 were excluded for the following reasons: significant alcohol intake (*n* = 80), positivity for HBsAg or anti-HCV (*n* = 35), and absence of laboratory results for noninvasive fibrosis indices (*n* = 104). Hence, 819 patients were enrolled in the present study (Fig. 1) and categorized into two parts. The first was a derivation group of patients who first visited the nephrology clinic between January 2011 and December 2014 (*n* = 567). The second part was a temporal validation group, including more recently recruited patients (between January 2015 and December 2016, *n* = 252) in the same institution [16]. The present study was conducted according to the ethical guidelines of the World Medical Association Declaration of Helsinki. The present study was approved by the institutional review board of Ewha Womans University Mokdong Hospital (approval No.: EUMC 2017–05–070-001), and the requirement for informed consent was waived because this was a retrospective study involving a review of electronic medical records. All data were de-identified and were reviewed upon approval.

### Data collection

The medical records of study participants were reviewed and data were collected, including medical history, drinking status, status of renal replacement therapy, and demographic characteristics, such as age and sex. Hypertension was defined as taking antihypertensives without regard to the actual measurement of blood pressure. Diabetes was defined as having a history of diabetes diagnosis. Dyslipidemia was defined as one or more reported prescriptions for a lipid-lowering agent. BMI was calculated as weight divided by the square of height (kg/m^2^). Laboratory data included blood cell count, serum aspartate aminotransferase (AST), serum alanine aminotransferase (ALT), total bilirubin, albumin, fasting plasma glucose (FPG), HbA1c, total cholesterol, TG, creatinine, and C-reactive protein obtained by standard clinical chemistry techniques. The triglyceride-glucose (TyG) index was calculated using the following formula: TyG  =  Ln [TG (mg/dL)  ×  FPG (mg/dL)/2] [17, 18]. The Hepatic Steatosis Index (HSI) score was calculated according to the following formula: HSI = 8 × ALT/AST ratio + BMI (+ 2, if diabetes; + 2, if female) [19]. The diagnosis of NAFLD was based on abdominal ultrasound in the absence of excessive alcohol consumption and other coexisting liver diseases. Abdominal ultrasound examinations were performed by a radiologist with more than 10 years of experience with abdominal ultrasound. High-resolution ultrasound (IU-22, Philips Healthcare, Bothell, WA, USA; HDI 5000, Philips Healthcare, Bothell, WA, USA) was used for examination by applying a 2–5 MHz convex transducer. Images were captured in a standard fashion with the patient in a supine position, and with the right arm raised above the head. Ultrasound diagnosis of fatty liver was defined as the presence of at least two of the following findings: (1) a diffuse increase of fine echoes in the liver parenchyma compared with the spleen or kidney parenchyma, (2) ultrasound beam attenuation, and (3) poor visualization of intrahepatic structures [20]. For each patient, the serum creatinine value was measured using the standardized glomerular filtration rate (GFR) method in the hospital laboratory department. Estimated glomerular filtration rate (eGFR) was then calculated from serum creatinine value using the Chronic Kidney Disease Epidemiology Collaboration (CKD-EPI) equation [21]. CKD was defined as eGFR < 60 ml/min/1.73 m^2^. CKD stage was defined by the KDIGO 2012 guideline as follows: stage 3a, eGFR = 45–59 mL/min/1.73 m^2^; stage 3b, eGFR = 30–44 mL/min/1.73 m^2^; stage 4, eGFR = 15–29 mL/min/1.73 m^2^; and stage 5, eGFR < 15 mL/min/1.73 m^2^ [22]. Among subjects with NAFLD, we calculated noninvasive scores for liver fibrosis, including NAFLD fibrosis score, BARD score, AST/ALT ratio, and FIB-4 score. NAFLD fibrosis score was calculated as follows: − 1.675 + 0.037 × age (year) + 0.094 × BMI (kg/m^2^) + 1.13 × impaired fasting glycemia/diabetes (yes = 1, no = 0) + 0.99 × AST/ALT ratio − 0.013 × platelet count (× 10^9^/L) − 0.66 × albumin (g/dL) [23]. The BARD score is composed of three variables: AST/ALT ratio ≥ 0.8 (2 points), BMI ≥28 kg/m^2^ (1 point), and the presence of diabetes (1 point). A BARD score of < 2 has a strong negative predictive value for advanced hepatic fibrosis associated with NAFLD [24]. FIB-4 score was calculated as age [years] × AST [IU/L]/platelet count [expressed as platelets × 10^9^/L] × (√ALT [IU/L]) [25, 26].

### Statistical analysis

Baseline characteristics are presented as mean ± standard deviation for normally distributed continuous variables, and as median with interquartile ranges for continuous variables with a skewed distribution. Discrete variables are summarized as numbers with percentages. To compare baseline characteristics by the presence of NAFLD, we used the Student t-test or Mann-Whitney U-test, as appropriate. Skewed distribution variables were transformed into natural logarithms and used for further analysis. Categorical variables were compared using the chi-square and Fisher’s exact tests. The entire cohort was divided into two parts to derive and validate a risk prediction model. In the derivationgroup (data collected between 2011 and 2014), simple logistic regression analysis was used to identify relevant features associated with the presence of NAFLD, and variables with *P* < 0.05 were subsequently included in the multivariate analysis. The selected variables for the logistic regression analysis included clinical characteristics, laboratory parameters, and noninvasive fibrosis markers. Forward and backward stepwise selection procedures were sequentially used to select the best-fitted model. For the selected model, the predictive performance was confirmed based on the AUROCs. We examined goodness of fit by comparing the observed response with the expected response, estimated from the risk score with a Hosmer-Lemeshow test. An optimal cutoff value was determined based on Youden’s index to maximize both sensitivity and specificity for risk stratification. Based on the cutoff value, we calculated sensitivity, specificity, positive predictive value, and negative predictive value. The model was internally validated and optimized with 1000 times bootstrapping. Finally, using the data collected between 2015 and 2016, a temporal validation of the performance of the prediction model was performed as described. Subgroup analysis for patients with NAFLD was conducted to evaluate the severity of liver fibrosis. A two-sided *P-*value < 0.05 was considered statistically significant. All statistical analyses were performed using SAS software ver. 9.4 (SAS Institute, Cary, NC, USA).

## Results

### Baseline patient characteristics

This study enrolled 819 patients with CKD. Table 1 summarizes the baseline characteristics of the derivation (*n* = 567) and validation (*n* = 252) groups. In the derivation group (data collected between 2011 and 2014), the mean age was 64.7 ± 15.3 years, and the prevalence of NAFLD was 15.7%. Patients in the NAFLD group had a significantly higher body mass index (BMI; 27.2 ± 16.3 vs. 24.1 ± 3.6 kg/m^2^), triglyceride (192 vs. 119 mg/dL), and total cholesterol (178.7 ± 42.4 vs. 163.2 ± 44.9 mg/dL) than those in the non-NAFLD group (all *P* < 0.05). Although the prevalence of hypertension and diabetes was similar between the groups, dyslipidemia was significantly higher in the NAFLD group (*P* < 0.001). For biochemical markers of kidney function, patients in the NAFLD group had a lower serum creatinine level (1.7 vs. 2.4 mg/dL) and a higher median estimated glomerular filtration rate (eGFR; 40.1 vs. 25.6 ml/min). Stages of CKD were categorized according to eGFR as follows: G3a (45–59 ml/min), *n* = 102 (18.0%); G3b (30–44 ml/min), *n* = 165 (29.1%), G4 (15–29 ml/min), *n* = 139 (24.5%); and G5 (< 15 ml/min), *n* = 161 (28.4%). Patients in the NAFLD group were more likely to have higher plasma fasting glucose levels, HbA1c, and TyG index compared to those in the non-NAFLD group.

**Table 1:** Baseline characteristics of CKD patients in the derivation and validation group

Variable	Derivation group				Validation group			
	All (*n* = 567)	Control (*n* = 478)	NAFLD (*n* = 89)	*P*	All (*n* = 252)	Control (*n* = 201)	NAFLD (*n* = 51)	*P*
Age (year)	64.7 ± 15.3	65.0 ± 15.1	63.0 ± 16.3	0.243	64.6 ± 14.8	65.9 ± 15.2	59.4 ± 12.1	0.005
Male	317 (55.9%)	253 (52.9%)	64 (71.9%)	< 0.001	165 (65.5%)	124 (61.7%)	41 (80.4%)	0.012
BMI (kg/m^2^)	24.6 ± 3.8	24.1 ± 3.6	27.2 ± 16.3	< 0.001	25.4 ± 3.8	24.7 ± 3.6	27.8 ± 3.7	< 0.001
Hypertension	459 (81.0%)	383 (80.1%)	76 (85.4%)	0.245	211 (83.7%)	169 (84.1%)	42 (82.4%)	0.765
Diabetes	233 (41.1%)	195 (40.8%)	38 (42.7%)	0.738	94 (37.3%)	74 (36.8%)	20 (39.2%)	0.752
T1DM	10 (4.2%)	9 (4.6%)	1 (2.6%)		2 (2.1%)	2 (2.7%)	0 (0.0%)	
T2DM	223 (95.8%)	186 (95.4%)	37 (97.4%)		92 (97.9%)	72 (97.3%)	20 (100.0%)	
Diabetic intervention								
Diet	65 (27.9%)	63 (32.3%)	2 (5.3%)		28 (29.8%)	27 (36.5%)	1 (5.0%)	
Oral hypoglycemic agent	78 (33.5%)	54 (27.7%)	24 (63.2%)		46 (49.0%)	36 (48.6%)	10 (50.0%)	
Insulin	90 (38.6%)	78 (40.0%)	12 (31.5%)		20 (21.2%)	11 (14.9%)	9 (45.0%)	
Dyslipidemia	71 (12.5%)	52 (10.9%)	19 (21.3%)	0.006	46 (18.3%)	33 (16.5%)	13 (25.5%)	0.139
Creatinine (mg/dL)	2.3 (1.6–4.4)	2.4 (1.7–4.7)	1.7 (1.5–2.5)	< 0.001	1.8 (1.4–2.4)	1.8 (1.4–2.5)	1.7 (1.4–2.3)	0.321
eGFR (ml/min)	28.0 (13.2–40.6)	25.6 (11.3–38.2)	40.1 (25.9–50.4)	< 0.001	36.0 (23.9–45.8)	35.7 (21.9–45.0)	40.0 (28.3–50.3)	0.036
eGFR category (KDIGO)								
G3a (45–59)	102 (18.0%)	63 (13.2%)	39 (43.8%)	< 0.001	70 (27.8%)	51 (25.4%)	19 (37.3%)	0.102
G3b (30–44)	165 (29.1%)	144 (30.1%)	21 (23.6%)		89 (35.3%)	73 (36.3%)	16 (31.4%)	
G4 (15–29)	139 (24.5%)	117 (24.5%)	22 (24.7%)		62 (24.6%)	48 (23.9%)	14 (27.5%)	
G5 (< 15)	161 (28.4%)	154 (32.2%)	7 (7.9%)		31 (12.3%)	29 (14.4%)	2 (3.9%)	
RRT	31 (5.5%)	30 (6.3%)	1 (1.1%)	0.070	12 (4.8%)	12 (6.0%)	0 (0.0%)	0.133
EPO use	208 (36.7%)	201 (42.1%)	7 (7.9%)	< 0.001	42 (16.7%)	40 (19.9%)	2 (3.9%)	0.006
White blood cell	7.0 (5.5–8.4)	6.8 (5.4–8.3)	7.5 (6.4–8.6)	0.008	6.7 (5.6–8.0)	6.6 (5.5–7.9)	7.0 (6.2–8.3)	0.090
Hemoglobin	10.9 ± 2.3	10.6 ± 2.1	12.7 ± 2.4	< 0.001	12.2 ± 2.2	11.8 ± 2.1	13.8 ± 1.8	< 0.001
Platelet	219.4 ± 71.7	216.9 ± 72.0	232.9 ± 69.2	0.053	233.8 ± 64.5	230.1 ± 64.9	248.5 ± 61.3	0.074
AST (IU/L)	20 (16–26)	20 (16–25)	22 (18–28)	0.009	21 (17–27)	21 (17–25)	24 (20–28)	0.003
ALT (IU/L)	17 (12–24)	16 (11–22)	21 (16–33)	< 0.001	17 (12–24)	16 (12–21)	25 (19–39)	< 0.001
Total. bilirubin (g/dL)	0.5 (0.3–0.6)	0.4 (0.3–0.6)	0.6 (0.4–0.8)	< 0.001	0.6 (0.4–0.7)	0.5 (0.4–0.7)	0.7 (0.5–0.9)	0.011
Albumin (mg/dL)	3.6 ± 0.6	3.5 ± 0.6	3.9 ± 0.5	< 0.001	3.8 ± 0.5	3.8 ± 0.4	3.8 ± 0.7	0.834
CRP	0.6 (0.1–4.3)	0.6 (0.1–4.1)	0.6 (0.2–5.3)	0.228	0.1 (0.1–0.4)	0.1 (0.1–0.2)	0.3 (0.1–0.4)	0.100
Triglyceride (mg/dL)	125 (91–182)	119 (86–164)	192 (134–270)	< 0.001	121 (88–170)	113 (84–156)	170 (112–237)	< 0.001
Total cholesterol (mg/dL)	165.7 ± 44.9	163.2 ± 44.9	178.7 ± 42.4	0.003	170.7 ± 46.6	168.4 ± 40.4	179.4 ± 65.0	0.257
Fasting Glucose (mg/dL)	101 (91–128)	101 (90–126)	106 (95–139)	0.018	102 (93–116)	101 (92–114)	108 (96–121)	0.029
HbA1c (%)	6.4 (5.9–7.5)	6.3 (5.8–7.4)	6.8 (6.3–8.4)	0.005	6.1 (5.6–6.9)	6.0 (5.6–6.8)	6.4 (5.7–7.4)	0.094
TyG index	8.9 ± 0.6	8.8 ± 0.6	9.3 ± 0.6	< 0.001	8.8 ± 0.6	8.7 ± 0.6	9.2 ± 0.7	< 0.001
HSI	35.9 (32.6–39.3)	35.6 (32.3–39.1)	36.8 (34.1–39.6)	0.017	36.8 (34.0–40.0)	36.9 (33.9–40.0)	36.4 (34.2–40.5)	0.855

Abbreviations: CKD, chronic kidney disease; NAFLD, nonalcoholic fatty liver disease; BMI, body mass index; T1DM, Type 1 diabetes mellitus; T2DM, Type 2 diabtes mellitus; eGFR, estimated glomerular filtration rate; KDIGO, kidney disease improving global outcomes; RRT, renal replacement therapy; EPO, erythropoietin; AST, aspartate aminotransferase; ALT, alanine aminotransferase; CRP, C-reactive protein; HbA1c, hemoglobin A1c;TyG index, triglyceride-glucose index; HSI, hepatic steatosis index

### Predictive factors for NAFLD in CKD patients

**Fig. 2:**
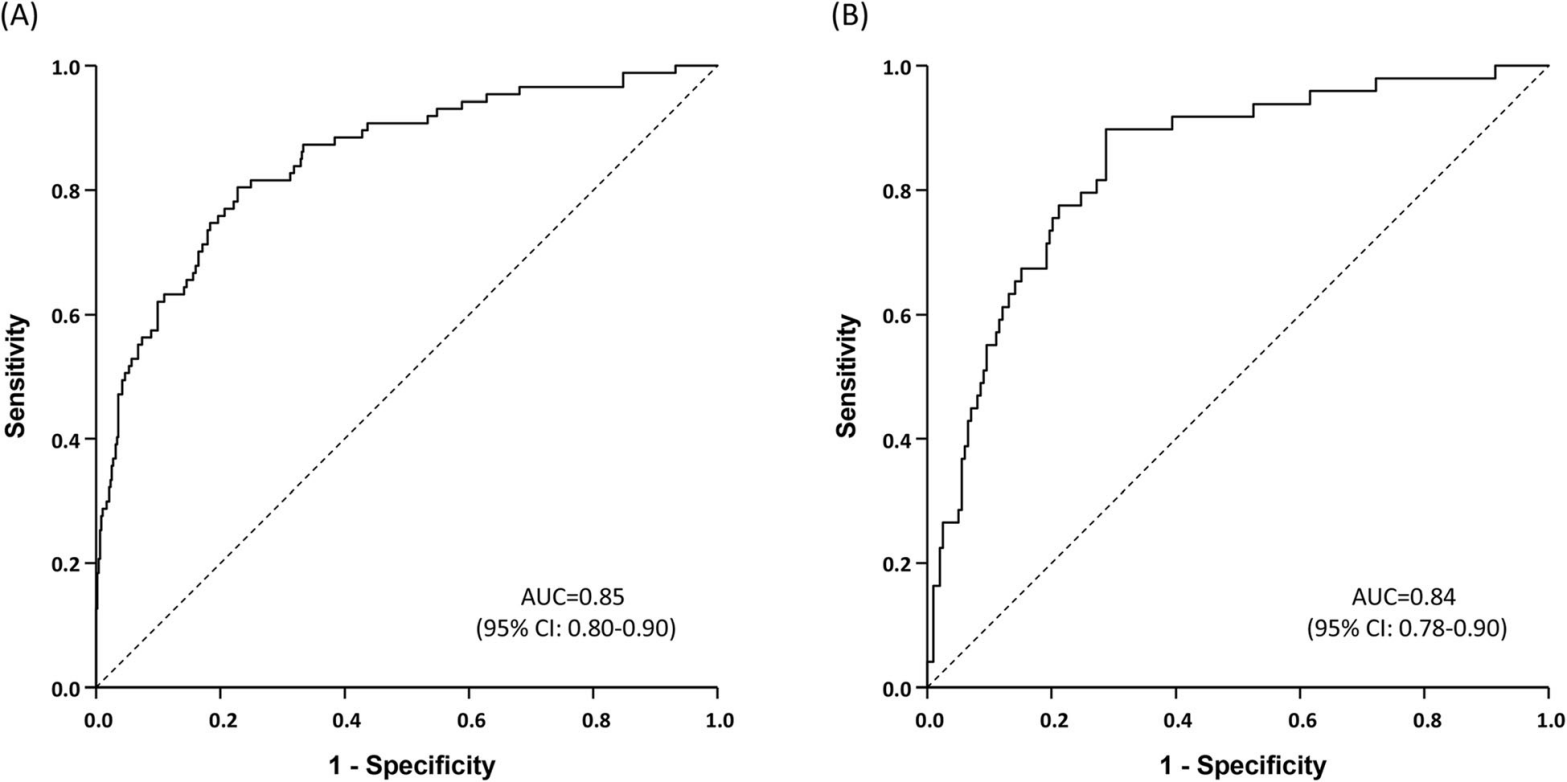
AUROCs for the prediction of NALFD in patients with CKD. (A) The AUROC was 0.850 (95% CI, 0.80–0.90) in the derivation group (*n* = 567). (B) The AUROC was 0.842 (95% CI, 0.78–0.90) in the validation group(*n* = 252). Abbreviations: AUROC, area under the receiver operating characteristic curve; NAFLD, nonalcoholic fatty liver disease; CKD, chronic kidney disease; CI, confidence interval

Multivariate logistic regression analyses were performed in the derivation group to identify relevant clinical and laboratory factors associated with the presence of NAFLD (Table 2). Predictors of NAFLD included higher values of BMI (odds ratio [OR], 3.247; 95% CI, 1.849–5.703; *P* < 0.001), hemoglobin (OR, 1.247; 95% CI, 1.084–1.434;*P* = 0.002), ALT (OR, 1.644; 95% CI, 1.034–2.616; *P* = 0.035), eGFR (OR, 3.538; 95% CI, 1.801–6.948; *P* < 0.001), and TyG index (OR, 4.903; 95% CI, 3.046–7.893; *P* < 0.001). A prediction model for the presence of NAFLD was derived based on these variables as follows: − 21.0935 + 0.2204 × hemoglobin + 1.590 × TyG + 0.4974 × log (ALT) + 1.263 × eGFR category (3.1 = 1; 3.2, 4, 5 = 0) + 1.1779 × BMI category (1 if BMI ≥ 25 kg/m^2^; 0 if BMI < 25 kg/m^2^). Using 0.146 as the cutoff value, the AUROC for the prediction of NAFLD in the derivation group was 0.850 (95% CI, 0.803–0.897) (Fig. 2a). The goodness of fit for this model was confirmed given the absence of a significant difference between the predicted and observed value (Hosmer-Lemeshow test for calibration: *P* = 0.760). Additionally, the sensitivity, specificity, accuracy, positive- and negative likelihood ratios were 0.805, 0.772, 0.393, and 0.956, respectively.

**Table 2:** Univariate and multivariate logistic regression analyses for factors associated with NAFLD in CKD patients

Variable	Univariate Analysis			Multivariate Analysis		
	Odds Ratio	95% CI	*P*	Odds Ratio	95% CI	*P*
Age (year)	0.991	0.977–1.006	0.243			
Male	2.277	1.387–3.738	0.001			
BMI (kg/m^2^)	1.242	1.164–1.326	< 0.001	3.247	1.849–5.703	< 0.001
Hypertension	1.450	0.772–2.721	0.248			
Diabetes	1.081	0.684–1.709	0.738			
Dyslipidemia	2.224	1.241–3.984	0.007			
Log Creatinine (mg/dL)	0.407	0.262–0.632	< 0.001			
eGFR (ml/min)	1.050	1.033–1.067	< 0.001	3.538	1.801–6.948	< 0.001
eGFR category (KDIGO)						
G3a (45–59)	Ref					
G3b (30–44)	0.236	0.128–0.432	0.533			
G4 (15–29)	0.304	0.166–0.557	0.573			
G5 (< 15)	0.073	0.031–0.173	< 0.001			
RRT	0.170	0.023–1.261	0.083			
EPO use	0.118	0.053–0.260	< 0.001			
Log White blood cell	2.017	1.067–3.815	0.031			
Hemoglobin	1.525	1.364–1.706	< 0.001	1.247	1.084–1.434	0.002
Platelet	1.003	1.000–1.006	0.054			
Log AST (IU/L)	1.626	1.060–2.494	0.026			
Log ALT (IU/L)	2.137	1.519–3.007	< 0.001	1.644	1.034–2.616	0.035
Log Total bilirubin (mg/dL)	2.874	1.812–4.558	< 0.001			
Albumin (g/dL)	3.458	2.127–5.621	< 0.001			
Log CRP	1.088	0.938–1.263	0.266			
Log Triglyceride (mg/dL)	7.109	4.176–12.104	< 0.001			
Total cholesterol (mg/dL)	1.007	1.002–1.012	0.003			
Log Fasting glucose (mg/dL)	2.387	1.236–4.609	0.009			
HbA1c (%)	9.220	2.046–41.552	0.004			
TyG index	4.330	2.874–6.522	< 0.001	4.903	3.046–7.893	< 0.001

Abbreviations: NAFLD, nonalcoholic fatty liver disease; CKD, chronic kidney disease; CI, confidence interval; BMI, body mass index; eGFR, estimated glomerular filtration rate; KDIGO, kidney disease improving global outcomes; RRT, renal replacement therapy; EPO, erythropoietin; AST, aspartate aminotransferase; ALT, alanine aminotransferase; CRP, C-reactive protein; HbA1c, hemoglobin A1c; TyG index, triglyceride-glucose index

### Validation of predictive performance and subgroup analyses

The validation group comprised of 252 consecutive patients (Fig. 1, Table 1). Their mean age was 64.6 ± 14.8 years, and the prevalence of NAFLD was 20.2%. The mean BMI was 25.4 kg/m^2^, and the median eGFR was 36.0 ml/min. Patients were categorized according to their eGFR as follows: G3a, 70 (27.8%); G3b, 89 (35.3%), G4, 62 (24.6%); and G5, 31 (12.3%). Unlike the derivation group, there were no significant differences in baseline characteristics between the control and NAFLD patients, except for age. The model remained well-calibrated in the validation group (Hosmer-Lemeshow test: *P* = 0.324). The predictive performance of the model in the validation set improved using the same cutoff value (AUROC = 0.842; 95% CI, 0.803–0.897) (Fig. 2b); the sensitivity, specificity, accuracy, positive- and negative likelihood ratios were 0.898, 0.677, 0.407, and 0.964, respectively. Furthermore, subgroup analysis was performed in NAFLD patients to evaluate the severity of liver fibrosis (Table 3). NAFLD patients were categorized according to their eGFR as follows: G3a, 58 (41.4%); G3b, 37 (26.4%), G4, 36 (25.7%); and G5, 9 (6.5%).The increased prevalence of NAFLD in patients with preserved kidney function was statistically significant, but the NAFLD severity index according to eGFR dose was not significant.

**Table 3:** Comparison of renal function and NAFLD fibrosis scores in patients with NAFLD

Variable	Total (*n* = 140)					
eGFR category	NAFLD	NFS	BARD (0–1)	BARD (2–4)	AAR	FIB-4
G3a (45–59)	58 (41.4%)	−0.86 (1.725)	−0.86 (1.725)	1.09 (0.481)	1.09 (0.481)	1.84 (1.590)
G3b (30–44)	37 (26.4%)	−0.93 (1.429)	−0.93 (1.429)	1.08 (0.433)	1.08 (0.433)	1.63 (1.333)
G4 (15–29)	36 (25.7%)	−0.69 (1.375)	− 0.69 (1.375)	1.22 (0.496)	1.22 (0.496)	1.64 (1.551)
G5 (< 15)	9 (6.5%)	−0.36 (1.551)	−0.36 (1.551)	1.88 (0.567)	1.88 (0.567)	1.19 (0.574)
*P* for trend	<.0001	0.2838	0.2838		0.6292	0.1214

Abbreviations: NAFLD, nonalcoholic fatty liver disease; eGFR, estimated glomerular filtration rate; NFS, NAFLD fibrosis score; AAR, AST/ALT ratio; FIB-4, fibrosis-4

## Discussion

Unlike in the general population, there is scarcity of supporting evidence for the presence of NAFLD in CKD patients. Hence, given the prevalence and clinical significance of the disease, the necessity for a screening method for NAFLD in CKD patients has become an important unmet need [27, 28]. Our prediction model incorporating BMI, hemoglobin, eGFR, ALT, and TyG index as covariates showed good predictive performance regarding the presence of sonographic NAFLD in CKD patients. The prevalence of NAFLD was higher in patients with preserved kidney function, and they had more severe liver fibrosis than advanced CKD patients.

NAFLD and CKD share many cardiometabolic risk factors and mechanistic molecular pathways in their pathogeneses. Recent studies have shown that NAFLD was associated with a significantly increased risk for CKD development and progression even after adjustment for common risk factors [12, 29–31]. NAFLD may exacerbate hepatic insulin resistance and promote hypertension, inducing atherogenic dyslipidemia and inflammatory mediators in CKD [32–34]. However, despite several evidence linking NAFLD with CKD, bidirectional relationships between these two diseases still require clarification as regards pathophysiology and clinical features. Particularly, studies on the characteristics of NAFLD in CKD patients are limited.

In our CKD patients cohort, in line with the concept that NAFLD is the hepatic manifestation of the metabolic syndrome, the presence of NAFLD was associated with obesity and components of metabolic syndrome such as serum triglyceride and fasting glucose [35–37]. Insulin resistance contributes to the initial fat accumulation in hepatocytes and the progression of simple steatosis to steatohepatitis or cirrhosis [38]. Additionally, the TyG index, which is a marker of insulin resistance, was found to be positively related to NAFLD risk in the present study [17, 18]. As insulin resistance is an early alteration in CKD pathogenesis, the presence of NAFLD in relation to insulin resistance and metabolic risk factors in our CKD patients supports a potential bidirectional mechanistic link between CKD and NAFLD [39, 40]. However, the prevalence of diabetes or hypertension did not differ between the control and NAFLD groups both in the derivation and validation groups, suggesting that additional risk factors other than metabolic abnormalities might be relevant in the development of NAFLD in CKD patients.

Considering that 41% of the entire enrolled patients had diabetes, diabetic medications might have influenced on the natural course of NAFLD as an effect modifier. In particular, metformin has been suggested to delay disease progression in NAFLD. The protective effects of metformin on the onset of NAFLD in animal models were associated with changes in intestinal microbiota composition and lower translocation of bacterial endotoxins [41]. Several human studies have reported the positive effect of metformin on aminotransferases and/or liver histology in patients with NASH [42, 43]. However, despite an improvement in serum aminotransferases and insulin resistance, there is a mixed conclusion in the overall results of human studies in that metformin does not significantly improve liver histology [41, 44]. In the latest practice guidelines for NAFLD, metformin is not recommended for the treatment of NAFLD patients [45]. Further researches are needed to clarify the effects of diabetic medications on the development of NAFLD and outcomes of CKD patients with NAFLD.

Despite the strong association of lipid profile on the presence of NAFLD as shown in Table 2, only 12.5% of the study population had been prescribed lipid-lowering agents (LLA). In NAFLD patients, insulin resistance promotes the overproduction and secretion of low-density lipoprotein [46, 47]. LLAs should be considered as a therapeutic option in patients with NAFLD and dyslipidemia to prevent adverse cardiovascular outcomes [48]. However, several studies among patients undergoing dialysis did not show the benefits of LLA on major vascular events [49–51]. A recent meta-analysis reported a reduction of major vascular events with LLA treatments as eGFR declined [52]. Given the importance of dyslipidemia on the cardiovascular outcome in NAFLD and a higher risk of NAFLD in earlier CKD in our study, initiation of LLA therapy in patients with preserved renal function needs to be underscored because of the smaller risk reduction in advanced CKD.

Serum hemoglobin was another independent risk factor for the presence of NAFLD in CKD patients in the present study. Because a significant proportion of patients used erythropoietin (derivation group, 36.7%; validation group, 16.7%), we checked the potential interaction between erythropoietin use and hemoglobin as a risk factor for NAFLD. No significant interaction was revealed. Several previous studies have suggested the putative role of hemoglobin in the development of NAFLD in the non-CKD population. A Chinese study reported that a higher hemoglobin level was an independent predictor of NAFLD in non-obese patients [53, 54]. Another study showed that serum hemoglobin levels were significantly associated with incidental metabolic syndrome and NAFLD in men [55]. Because higher hemoglobin levels cause increased blood viscosity, blood glucose supply to the muscle reduces as the blood flow slows down [56]. Insulin resistance appears to be mediated by reduced intracellular glucose translocation [26]. Moreover, insulin and insulin-like growth factor may stimulate marrow red blood cell generation, which further increases blood concentration and blood viscosity [57]. These data support the association between higher hemoglobin levels and increased risk of NAFLD in our CKD population. One caveat is the lower absolute values of hemoglobin of our study population compared to previous reports [53–55]. However, the present study exclusively enrolled CKD patients who frequently experienced anemia due to a deficit of erythropoietin. The relevance of hemoglobin on the development of NALFD seems to be maintained in CKD patients regardless of the absolute level of hemoglobin according to our results and the plausible mechanisms described above. More studies are needed to prove the pathophysiologic role of hemoglobin in the development of NAFLD.

Serum ALT levels were significantly higher in NAFLD patients with CKD. The mechanism of NAFLD development is explained by insulin resistance, which activates lipolysis resulting in the accumulation of non-esterified fatty acids [58]. This enhanced fat accumulation in the liver is directly toxic to hepatocytes. Hence, increased serum ALT levels seem to be due to NAFLD, rather than the cause of NAFLD. Although serum ALT is widely used as a surrogate marker for NAFLD in practice or in epidemiological studies, the use of increased ALT level alone may underestimate the severity of liver injury because normal or mildly elevated ALT may be related to significant histological abnormalities [59]. At least, ALT seems useful as a component of the prediction model for the presence of NAFLD in CKD patients according to our results.

In our CKD population, NAFLD was more frequently observed in patients with relatively preserved renal function. This was an unexpected finding because the severity of hepatic steatosis in NAFLD was associated with increased CKD risk and severity in a previous study [60]. Given the strong relationship between NAFLD and early kidney dysfunction in a recent study in obese children [30], we speculated that milder CKD was more likely to be related to NAFLD in its pathogenesis along with the increasing prevalence of NAFLD, whereas, advanced CKD might have resulted from etiologies other than NAFLD. Although a causal relationship between CKD and NAFLD requires clarification in prospective studies, the results of the present study suggest the necessity of NAFLD screening in patients with early-stage CKD to prevent the simultaneous progression of NAFLD as a severe comorbidity, which is frequently underdiagnosed due to its insidious nature.

Caution is needed to interpret the results of the present study due to the following limitations. First, although we found relevant risk factors for the presence of NAFLD, which are biologically plausible, the retrospective nature of the study could have affected the results, especially concerning a potential selection bias. However, we attempted a temporal validation, and the performance of our prediction model was maintained in the validation. Second, the lack of histological or radiological data such as ultrasound-based or magnetic resonance-based elastography prevented the refined categorization of NAFLD severity. Instead, we employed widely used and validated serum marker-based indices such asNAFLD fibrosis score and FIB-4 [61]. Therefore, well-designed and adequately powered prospective and randomized clinical studies of patients with biopsy-proven NAFLD are needed. Again, although validated, our results were derived from a cross-sectional analysis, which warrants further investigation with longitudinal data to identify a causal or bidirectional relationship between CKD and NAFLD. Furthermore, CKD was defined based on eGFR using CKD-EPI formula, in which the use of creatinine as a filtration marker provided inherent limitations especially for obese patients [62, 63].

## Conclusions

In conclusion, this study identified relevant predictors of the presence of NAFLD in CKD patients. The predictors of the presence of NAFLD showed an accurate predictive performance, which was confirmed in a temporal validation. Given the absence of specific noninvasive predictive tools for the presence of NAFLD as a comorbidity of CKD patients to date, screening for NAFLD based on these risk factors seems reasonable in CKD patients. This is because CKD patients are prone to liver-related morbidity or mortality, and all-cause or cardiovascular mortality on top of underlying CKD and other related outcomes. These results need further validation, including assessment for liver disease severity and liver- and kidney-related outcomes in a prospective setting.

## Data Availability

The dataset used in the analysis is available with the corresponding author and will be released on request.

## Abbreviations

ALT: Alanine aminotransferase
Anti-HCV: Hepatitis C virus antibodies
AST: Aspartate aminotransferase
AUROC: Area under the receiver operating characteristic curve
BMI: Body mass index
CKD: Chronic kidney disease
CKD-EPI: Chronic kidney disease epidemiology collaboration
eGFR: Estimated glomerular filtration rate
FIB-4: Fibrosis-4
FPG: Fasting plasma glucose
HBsAg: Hepatitis B surface antigen
HIS: Hepatic steatosis index
KDIGO: Kidney disease improving global outcomes
LLA: Lipid-lowering agent
NAFLD: Nonalcoholic fatty liver disease
OR: Odds ratio
TyG: Triglyceride-glucose
